# Spontaneous Hemoperitoneum due to Rupture of Uterine Varicose Veins during Labor Successfully Treated by Percutaneous Embolization

**DOI:** 10.1155/2014/580384

**Published:** 2014-07-09

**Authors:** Rebeca Díaz-Murillo, Pablo Tobías-González, Sara López-Magallón, Fernando Magdaleno-Dans, José L. Bartha

**Affiliations:** Division of Maternal Fetal Medicine, Department of Obstetrics and Gynecology, University Hospital La Paz, Paseo de la Castellana 261, 28046 Madrid, Spain

## Abstract

Hemoperitoneum during pregnancy is a rare but potentially lethal clinical condition. Improvements in antenatal and intrapartum care, especially in surgical and anesthetic techniques, have reduced maternal mortality; perinatal mortality remains very high (31%). Treatment is based on the systemic correction of hypovolemia and immediate surgery via laparotomy or laparoscopy in cases in the first trimester of pregnancy for hemostatic purposes. Sometimes, hysterectomy is needed. A 35-year-old Asiatic primigravid woman at 37 weeks' gestation with otherwise uneventful pregnancy came to the hospital referring abrupt-onset lumbar and abdominal pain. A bleeding uterine superficial varicocele of about 7 cm was found on the left uterine horn during Caesarean section. Interventional radiologic embolization of both uterine arteries was successfully performed. Posterior evolution of the patient was favorable. Percutaneous vascular embolization of the uterine arteries is an effective alternative treatment for many obstetrical and gynecological causes of bleeding. The main advantage of this technique is the low rate of serious complications and the preservation of reproductive function. To our knowledge, this is the first case of spontaneous intrapartum hemoperitoneum treated with this technique. An early diagnosis and a rapid indication of this therapeutic option are essential. Hemodynamic stability is needed to decide this conservative management.

## 1. Background

Hemoperitoneum during pregnancy secondary to rupture of superficial uterine varicose veins is a rare but potentially lethal clinical condition [1]. According to a reported series in 1950, maternal mortality may reach until 49% [2]. However, improvements in antenatal and intrapartum care, especially in surgical and anesthetic techniques, have reduced this rate until 3.4% in 1987. Furthermore, perinatal mortality remains very high reaching 31% in this last series. Most of these cases occur antenatally (61%) especially during the third trimester of pregnancy and more rarely during labor (18%) or puerperium (21%) [3].

Spontaneous rupture of uterine veins is very uncommon. The incidence is 1/10000 births. In the English literature, around 150 cases had been reported until now. The cause is still unknown. The most common places where it happens are: broad ligament (78.3%), the back of the uterus (18.3%), and the front face of the uterus (3.3%) [4].

Early diagnosis is essential to prevent mortality and a late diagnosis has been clearly associated with a poor prognosis for both the mother and the infant. Diagnosis of hemoperitoneum before surgery is made in 37% of cases and this rate falls until only 4% in cases of rupture of uterine vessels [3]. It may be extremely difficult when this rupture occurs during labor because the presence of contractions may mask the pain associated with hemoperitoneum which causes a delay in diagnosis.

Typically, symptoms include acute abdominal pain associated with hypovolemia and low maternal hemoglobin levels [5–9]. Physical examination may show signs of peritoneal irritability or pelvic mass [5, 8]. Diagnostic confirmation may be done by using ultrasound. This technique allows us to corroborate the presence of fluid in abdominal cavity and to rule out the presence of placental abruption [8]. In this case, a poorly echogenic area can be seen in the placenta but it presents low sensibility.

Classically, treatment is based on the systemic correction of hypovolemia and immediate surgery via laparotomy or laparoscopy in cases in the first trimester of pregnancy for hemostatic purposes [6]. In cases at term usually Cesarean section is performed and in those cases in which the focus of the hemorrhage is not clear sometimes hysterectomy is needed [5, 8–10].

We report a case of spontaneous hemoperitoneum during labor due to ruptured uterine varicocele successfully treated with radiological embolization of both uterine arteries.

## 2. Case Report

A 35-year-old Asiatic primigravid woman at 37 weeks' gestation with otherwise uneventful pregnancy came to the Hospital referring abrupt-onset lumbar and abdominal pain. Cardiotocography showed a reassuring trace and the presence of low intensity uterine contractions. Ultrasound revealed a normal fetus in breech presentation, normal placenta, and normal amniotic fluid volume. On examination the cervix was long and closed and there was no evidence of external bleeding. Maternal hemoglobin at admission was 11.5 g/dL and there were no signs of hypovolemia.

**Figure 1:**
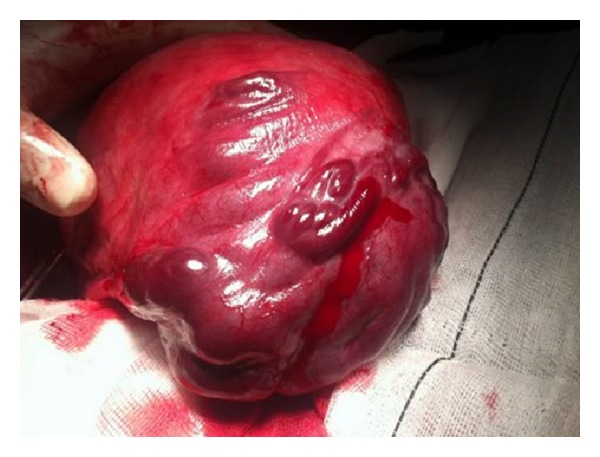
A bleeding uterine superficial varicocele of about 7 cm was found on the left uterine horn.

The patient was admitted for observation. Subsequent cardiotocography showed a mild fetal tachycardia while uterine contractions remained at low frequency and intensity. However the distressing back pain continued increasing. It just partially ameliorated by sitting and worsened with recumbency. Because of breech presentation, the presence of symptoms and gestational age a Cesarean section was indicated. After laparotomy a moderate hemoperitoneum was seen in the abdominal cavity. No bleeding point was evident. After fetal extraction and deliver of the placenta, the uterus was exteriorized. A bleeding uterine superficial varicocele of about 7 cm was found on the left uterine horn (Figure 1). Uterine wall was extremely thin at this area. There were numerous and friable bleeding points connecting with the left broad ligament. After a detailed examination of the abdominal cavity no other hemorrhagic area was located.

For treatment, local excision was not considered due to the extension of the injured area. Because the patient was hemodynamically stable and had future pregnancy aspirations, hysterectomy was not selected as the first therapeutical option. Surgical artery ligation was rejected because it was not technically possible to do and it is less effective than embolization due to the rich arterial collateral network of the pelvis. A procoagulant device (TachoSil Nycomed Pharma) was placed on the bleeding area and interventional radiologic embolization of both uterine arteries was successfully performed.

**Figure 2:**
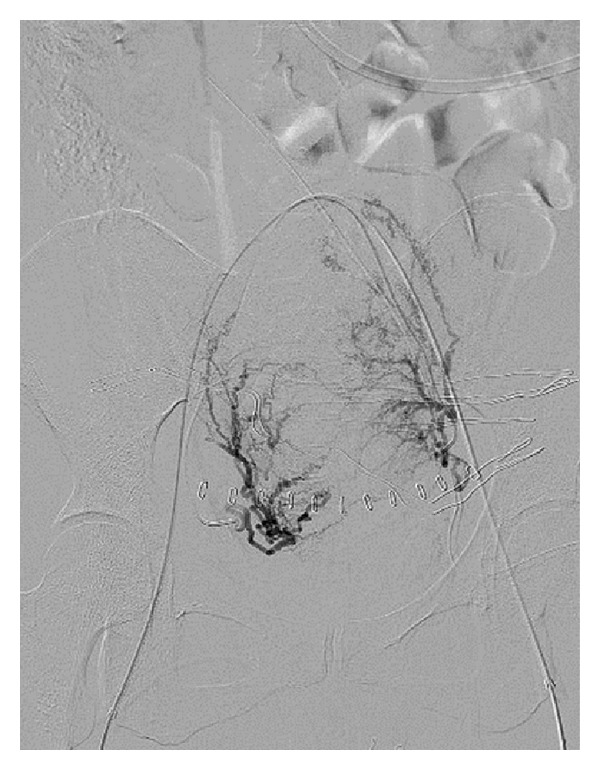
Arteriography of both uterine arteries before embolization.

The access way for the embolization was both common femoral arteries. After selective catheterization of both uterine arteries, a uterus with prominent arteries was observed. The left horn was more vascularized, withaout signs of extravasation (Figure 2). It was proceeded to embolization with polyvinyl alcohol particles of 500–700 microns.

Posterior evolution of the patient was favorable. Hemoglobin level postoperatively was 10.3 g/dL and no blood transfusion was required.

## 3. Discussion

Similar cases of spontaneous hemoperitoneum during pregnancy have been previously reported associated with spontaneous rupture of uterine vessels (veins or arteries), uterine varices, or uterine-ovarian vessels [10]. The influence of uterine contractions on the rupture of these vessels is still unknown. It is possible that myometrial activity can lead to an increased pressure in the uterine vessels previously dilated due to the physiological increased pressure in the iliac and inferior vena cava area caused by hormonal and anatomical reasons [7, 11].

The main causes of spontaneous hemoperitoneum due to obstetric reasons include rupture of interstitial ectopic pregnancy, uterine rupture due to placental accreta, rupture of uterine vessels, HELLP syndrome, or rupture of a rudimentary uterine horn. Among the nonobstetrics causes spontaneous rupture of umbilical maternal vein, rupture of splenic vein or arterial aneurysm, liver rupture (spontaneous hematoma), rupture of hemangioma, or liver metastases [7–10].

The particular tortuous, nonvalvular, and thin walled uterine veins anatomy under the high pelvic vein pressure during pregnancy are the factors predisposing for rupture. However, considering the rarity of the condition there should be some additional factors involved in its pathogenesis [3, 7, 9–11]. For example, it has been reported that the presence of endometriosis lesions [11], adhesions, vascular anomalies, previous uterine surgery, uterine fibroids, and multiparity may be considered as risk factors.

Concerning treatment, until now, the only possibility of improving the prognosis is to perform an urgent surgical procedure on the uterus or at least on the bleeding area. Percutaneous vascular embolization of the uterine arteries is an alternative and effective treatment for many obstetrical and gynecological causes of bleeding, such as uterine fibroids, adenomyosis, postpartum hemorrhage, abnormal placentation, and even ectopic pregnancy. The main advantage of this technique is the low rate of serious complications and the preservation of reproductive function. In order to integrate this method into clinical practice, it is necessary to know indications, vascular anatomy, the technical procedure, and the risks and benefits [12–14].

The main complications related to the arterial embolization include pulmonary embolism, infarction, transient ovarian failure, bladder wall necrosis, vaginal fistula, neurological damage, perforation, or occlusion of external iliac artery [15]. It has been described that the postembolization syndrome characterized by pain, fever, nausea, and leukocytosis immediately after the procedure reported in 50% of the patients. Uterine necrosis or rupture, sepsis, abscess, and ischemia are rare. Those risks are minimized by knowledge of the vascular anatomy and meticulous attention to embolization technique [16].

Pelvic arterial embolization plays an important role as a treatment option to control and prevent pregnancy related hemorrhage, which has been established to be safe and effective. Unilateral embolization carries a risk of secondary recanalization of bleeding and can continue through transpelvic vascular supply. For this reason, the technique should be a bilateral selective of the internal iliacs and uterine arteries. If the operator is unable to select the uterine artery, embolization of the anterior iliac branch is acceptable [15, 17]. Visualization of extravasation is not necessary for performing the embolization as this is more often not seen [15].

To our knowledge, this is the first case of spontaneous intrapartum hemoperitoneum treated with this technique. For that, it is essential an early diagnosis and a rapid indication of this therapeutic option. Hemodynamic stability is needed to decide this conservative management.
